# Supplementary material for: Prediction of Cyclosporin-Mediated Drug Interaction Using Physiologically Based Pharmacokinetic Model Characterizing Interplay of Drug Transporters and Enzymes
Source: Int J Mol Sci. 2020 Sep 24;21(19):7023. doi: 10.3390/ijms21197023 (PMC7582433; doi:10.3390/ijms21197023)
Supplement: Supplementary file 1 [file ijms-21-07023-s001.pdf]

Supplementary Material

Table S1. Calculation of SF

Drug	Reference of Obs data	SF	Mean
CsA	[1]	0.01	0.01
	[2]	0.01	
Flu	[3]	23	21
	[3]	22	
	[4]	20	
Sim	[5]	25.15	25
	[6]	25.9	
	[7]	25.28	
Lov	[8]	2.11	4
	[3]	4.21	
	[9]	6.08	

The calculated SF of cyclosporine, fluvastatin, simvastatin and lovastatin in Table 3 were estimated from the software based on their pharmacokinetic data showed in Table S1.

The Pred/obs figures for atorvastatin Report 2 and lovastatin Report 2 are showing in Figure S1. While the others Reports without figures were the ones which have no observed plasma concentration curve.

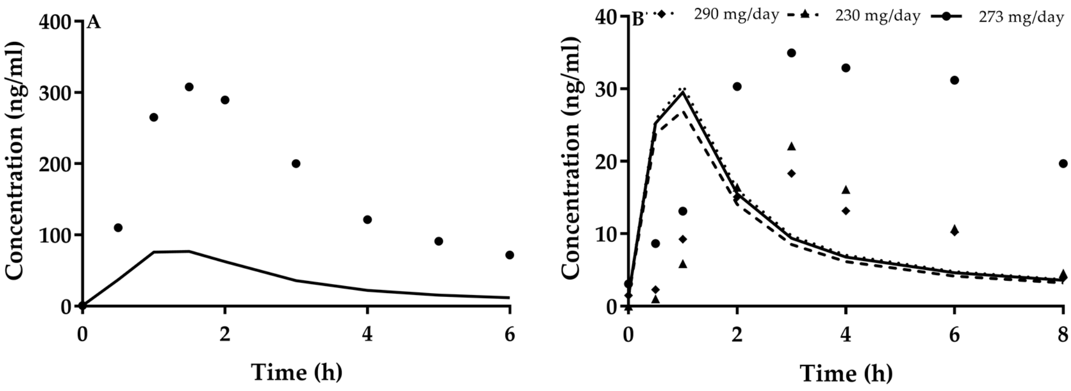

Figure S1. Predicted plasma concentrations (line) of the victim drugs agents using PBPK model and observed plasma concentrations (points) following oral administration to subjects when coadministrated with CsA (twice daily). **(A)** atorvastatin; **(B)** lovastatin. Observed data were cited from clinic reports. [10,11]

Reference

1. Guo Tao; Qin Haixu; Dongya, X. Pharmacokinetic and bioequivalence of three preparations of Cyclosporine A in Chinese healthy volunteers. Guang Zhou; pp. 5435-5443.
2. HE Jian-chang; FENG En-fu; ZH ANG Qing; YIN Yu-qin; HE Hong-jing; Gui-li., X. Study on bioequivalence of ciclosporin microemulsion oral solution in healthy volunteers. Pha rm Care & Res 2009, 9(2), 137-139.
3. Keskitalo, J.E.; Kurkinen, K.J.; Neuvonen, M.; Backman, J.T.; Neuvonen, P.J.; Niemi, M. No significant effect of ABCB1 haplotypes on the pharmacokinetics of fluvastatin,

pravastatin, lovastatin, and rosuvastatin. Br J Clin Pharmacol 2009, 68, 207-213, doi:10.1111/j.1365-2125.2009.03440.x.

4. Kalafsky, G.; Smith, H.T.; Choc, M.G. High-performance liquid chromatographic method for the determination of fluvastatin in human plasma. J Chromatogr 1993, 614, 307-313, doi:10.1016/0378-4347(93)80323-v.

5. Ayalasomayajula, S.; Han, Y.; Langenickel, T.; Malcolm, K.; Zhou, W.; Hanna, I.; Alexander, N.; Natrillo, A.; Goswami, B.; Hinder, M., et al. In vitro and clinical evaluation of OATP-mediated drug interaction potential of sacubitril/valsartan (LCZ696). J Clin Pharm Ther 2016, 41, 424-431, doi:10.1111/jcpt.12408.

6. Harvey, R.D.; Aransay, N.R.; Isambert, N.; Lee, J.S.; Arkenau, T.; Vansteenkiste, J.; Dickinson, P.A.; Bui, K.; Weilert, D.; So, K., et al. Effect of multiple-dose osimertinib on the pharmacokinetics of simvastatin and rosuvastatin. Br J Clin Pharmacol 2018, 84, 2877-2888, doi:10.1111/bcp.13753.

7. Chu, N.N.; Chen, W.L.; Xu, H.R.; Li, X.N. Pharmacokinetics and safety of ezetimibe/simvastatin combination tablet: an open-label, single-dose study in healthy Chinese subjects. Clin Drug Investig 2012, 32, 791-798, doi:10.1007/s40261-012-0013-5.

8. Yin, O.Q.; Mak, V.W.; Hu, M.; Fok, B.S.; Chow, M.S.; Tomlinson, B. Impact of CYP2D6 polymorphisms on the pharmacokinetics of lovastatin in Chinese subjects. Eur J Clin Pharmacol 2012, 68, 943-949, doi:10.1007/s00228-011-1202-5.

9. Tornio, A.; Vakkilainen, J.; Neuvonen, M.; Backman, J.T.; Neuvonen, P.J.; Niemi, M. SLCO1B1 polymorphism markedly affects the pharmacokinetics of lovastatin acid. Pharmacogenet Genomics 2015, 25, 382-387, doi:10.1097/FPC.0000000000000148.

10. Lemahieu, W.P.; Hermann, M.; Asberg, A.; Verbeke, K.; Holdaas, H.; Vanrenterghem, Y.; Maes, B.D. Combined therapy with atorvastatin and calcineurin inhibitors: no interactions with tacrolimus. Am J Transplant 2005, 5, 2236-2243, doi:10.1111/j.1600-6143.2005.01005.x.

11. Gullestad, L.; Nordal, K.P.; Berg, K.J.; Cheng, H.; Schwartz, M.S.; Simonsen, S. Interaction between lovastatin and cyclosporine A after heart and kidney transplantation. Transplant Proc 1999, 31, 2163-2165, doi:10.1016/s0041-1345(99)00295-x.
